# Habitat and local factors influence fish biomass recovery in marine protected areas

**DOI:** 10.1098/rspb.2024.2708

**Published:** 2025-07-09

**Authors:** Ella Clausius, Graham J. Edgar, Genevieve A. C. Phillips, Camille Mellin, Elizabeth Oh, Rick Stuart-Smith

**Affiliations:** ^1^ University of Tasmania Institute for Marine and Antarctic Studies, Hobart, Tasmania, Australia; ^2^ Centre for Marine Socioecology & Institute for Marine and Antarctic Studies, University of Tasmania, Hobart, Tasmania, Australia; ^3^ The Environment Institute and School of Biological Sciences, University of Adelaide, Adelaide, South Australia, Australia

**Keywords:** reef fish, conservation, marine protected area management, marine reserves

## Abstract

Well-designed and managed marine protected areas (MPAs) can have positive outcomes for reef biodiversity, but their effectiveness for conservation outcomes is also influenced by local environmental and anthropogenic factors. To assess the importance of local factors on MPA effectiveness, we compared field-collected data on total reef fish biomass from 922 sites inside and outside a network of 49 MPAs across temperate Australia using modelled predictions of biomass based on local biogenic habitat, physical environment and anthropogenic factors. We found fish biomass was 34% greater in fully protected MPAs in temperate Australia than predicted if they were openly fished, whereas biomass in partially protected MPAs was equivalent to fished sites. Local biogenic habitat and physical environmental features significantly shaped shallow reef biomass across large spatial scales but their effects did not differ between fished and fully protected MPA sites, providing reassurance that regional habitat change inside and outside MPAs will not greatly affect relative effect sizes. These findings affirm the role of fishing in shaping fish biomass on shallow reefs across broad spatial scales and underscore the importance of strict protection from fishing. Strategic MPA design and management should consider local conditions to refine expectations, optimize fish biomass recovery and enhance conservation outcomes.

## 1. Background

Marine protected areas (MPAs) are increasingly used as a management tool to reduce the impacts of human activity on reef biodiversity [1], but their effectiveness in protecting and restoring ecological communities is probably shaped by the larger seascape context in which they exist. Given adequate time [2,3], increases in reef fish richness, abundance and size (biomass) are possible inside well-placed [4,5], fully protected (i.e. no-take [6,7]), adequately resourced [8] and strictly enforced MPAs [6]. Yet heterogeneity in effect sizes even among well-designed, well-managed and well-enforced MPAs (e.g. [9]) suggests recovery of reef fish populations may be influenced by other factors, often unmeasured, that interact with protection [4,10]. In particular, the relative importance of local biogenic, physical environmental and anthropogenic factors in shaping the broader ecological outcomes of networks of MPAs is poorly known, even though such factors are known to be locally important for fish communities.

Temperate reef fish communities are highly dynamic, varying in abundance and biomass through space and time in response to a range of environmental and human factors across multiple scales. At a global scale, reef fish biomass, richness and abundance vary across latitudinal and longitudinal gradients, related to broad-scale ocean environmental conditions such as sea surface temperature (SST), annual temperature range and photosynthetically active radiation [6,11]. In fact, 63% of global patterns in reef fish community biomass can be explained on the basis of remotely sensed environmental variables and country-level socioeconomic factors [6]. At finer scales, communities are shaped directly and indirectly by complex and interacting top-down and bottom-up processes and pressures, including (i) anthropogenic pressures, such as recreational and commercial fishing [12]; (ii) biological factors, such as the condition and coverage of biogenic habitat [13,14]; and (iii) physical environment factors, such as wave exposure [15], depth [14] and habitat structure and complexity [16,17]. It is possible, therefore, that local conditions could influence the capacity of fish communities to respond to MPA protection, either positively or negatively. Of concern then, is the potential for long-term MPA success to be undermined by degrading conditions across the larger seascape.

Recovery of reef communities could be particularly hindered by deterioration in the condition of foundational biogenic habitat. Biogenic reef habitat, such as macroalgal kelps and corals, plays a critical role on temperate and tropical reefs, respectively, supporting higher levels of fish productivity and biodiversity through the provision of critical habitat, predator refuges and food sources for lower trophic guilds (e.g. herbivores, detritivores and corallivores [17–20]). The coverage and condition of both kelp [21] and coral habitats [22,23], however, are impacted worldwide by the direct and indirect effects of human activity, with potentially significant implications for the reef fish communities these habitats support [24,25]. While MPA protection may buffer reef fish communities against direct adverse effects associated with extreme environmental disturbance [26], they do not directly prevent the loss of macroalgal kelp or coral habitat [27,28]. Continued degradation of biogenic reef habitat could, therefore, hinder conservation efforts where they are needed most, highlighting the urgency to understand the role that local factors play in shaping MPA success.

Moreover, local conditions not only have the capacity to diminish the outcomes of individual MPAs but, through their collective impact, may undermine the ecological effectiveness of large MPA networks, with potentially significant consequences for international efforts to halt biodiversity loss. Since the adoption of the Convention on Biological Diversity’s Kunming-Montreal Global Biodiversity Framework (GBF) in 2022 [29], the international community is now working towards a revised target of 30% of the world’s marine area to be protected by MPAs and other effective means by 2030. Yet even if this ‘30 by 30’ target is achieved, declining environmental conditions and loss of foundational reef habitats worldwide may lead to overall reductions in the capacity of the global network to preserve and conserve marine biodiversity through time, irrespective of MPA design, management and enforcement. Considering current shortfalls in the global MPA network [30–34], understanding the relative importance of local factors in shaping outcomes across MPA networks is critical, not only for evaluating the current success of global area-based conservation efforts but also for predicting environmental outcomes into an increasingly uncertain future.

To understand the influence of local (i.e. 100 m^2^) factors on the ecological effectiveness of large MPA networks (i.e. spanning greater than 1000 km^2^), we quantified the relative importance of biogenic habitat, physical environment and anthropogenic factors in shaping shallow reef fish biomass across a network of 126 partially and fully protected zones from 49 MPAs, relative to protection and in the context of broader seascape variables. To do this, we contrasted field-collected observational data of total reef fish biomass from 922 sites spanning temperate Australian waters, here defined as south of 29° S, with modelled predictions of fish biomass using environmental variables to derive an estimate of differences in biomass at a given reef site. We then evaluated how biomass differences related to local biogenic reef habitat, physical environment and anthropogenic factors considered important for shaping shallow reef communities.

## 2. Methods

**Figure 1.**
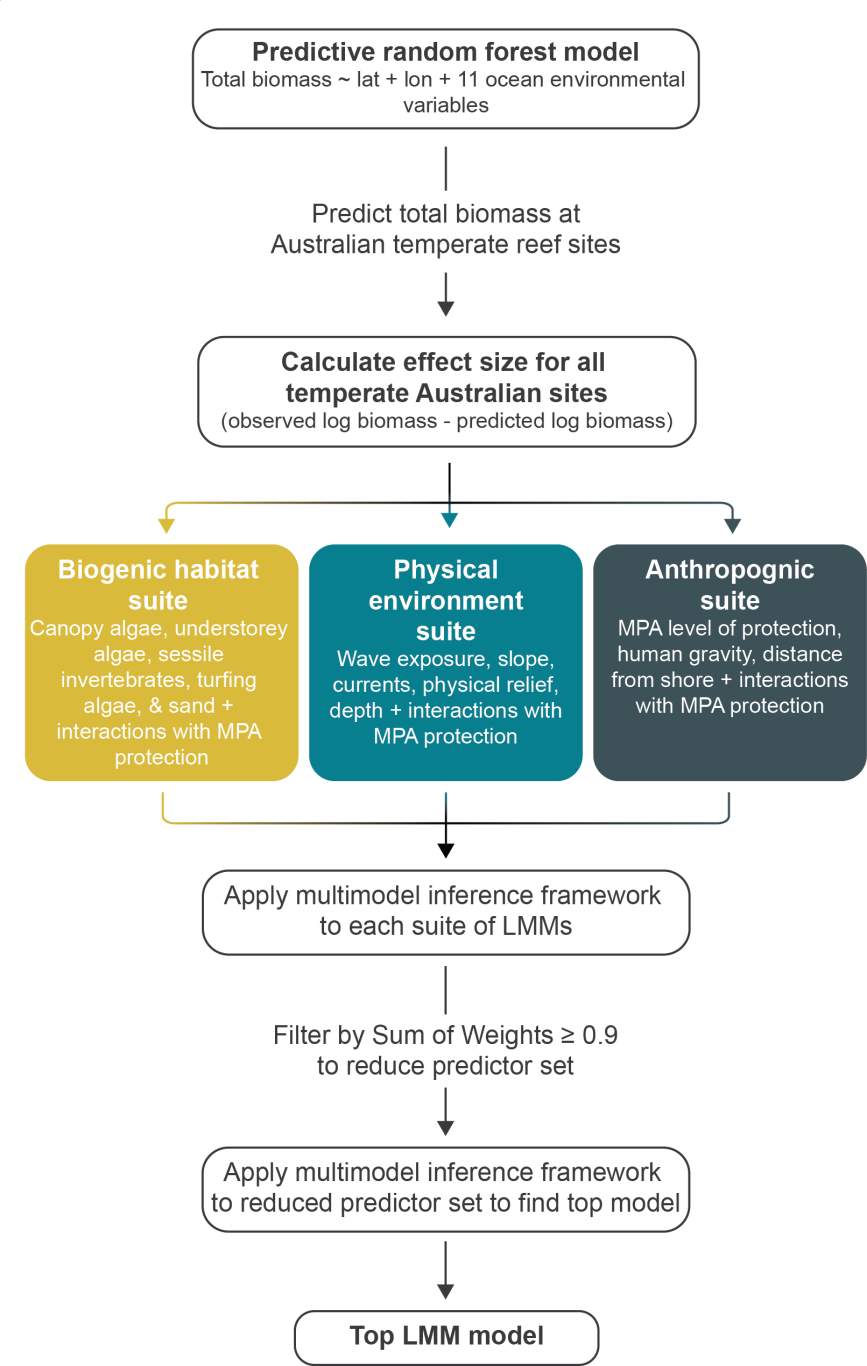
Overview of the study design used here to explore the relative importance of local biogenic, physical environment and anthropogenic factors in MPA effectiveness.

To address the aims of this study, we took a multi-staged approach, with an overview of the data and analyses used in each stage of the study detailed in figure 1.

### (a) Data

#### (i) Reef surveys

**Figure 2.**
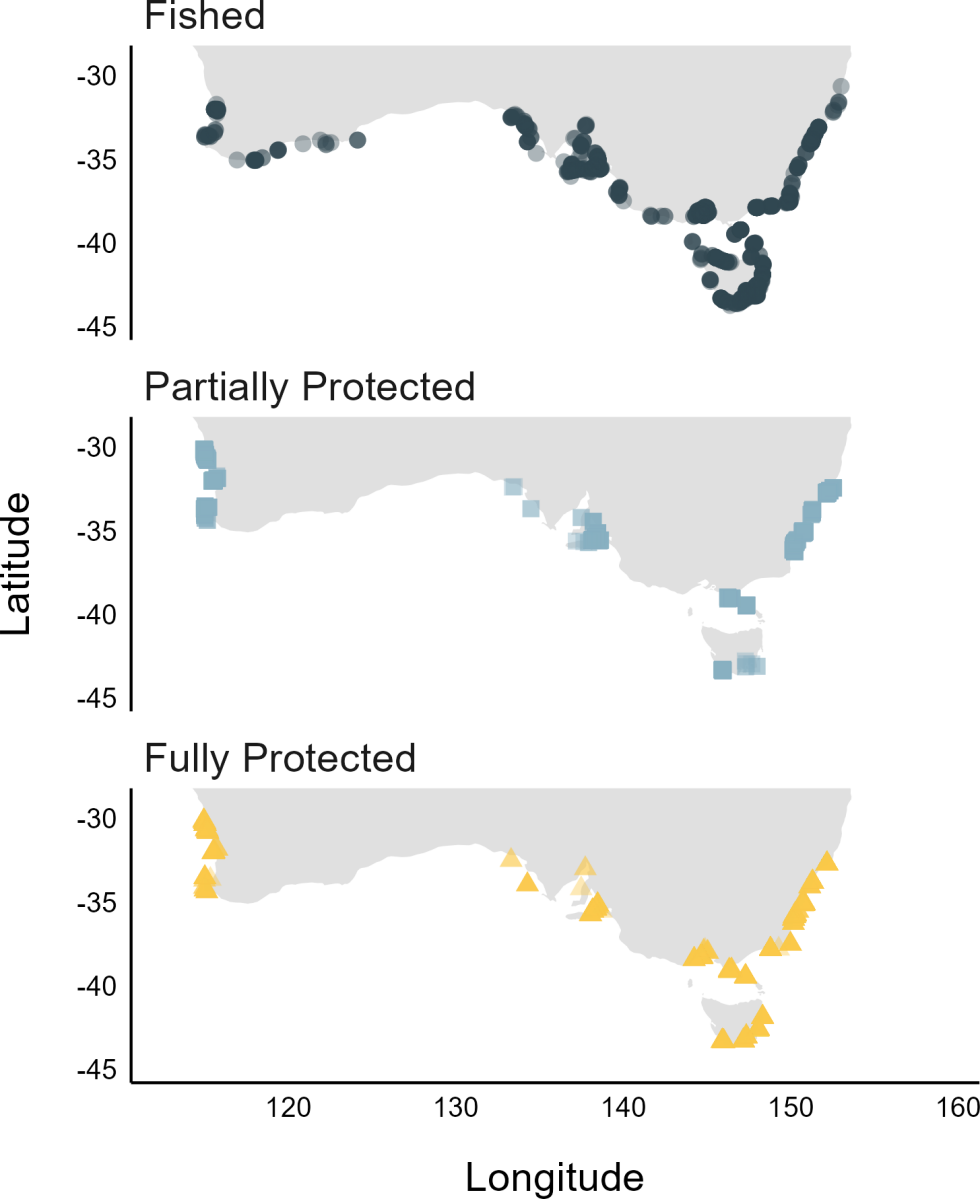
Temperate Australian shallow reef sites included in analysis, by level of protection against fishing.

The ecological data used in this study were collected by highly trained citizen science divers through the Reef Life Survey (RLS) programme [35], and scientific divers through the Australian Temperate Reef Collaboration (ATRC) and Parks Victoria monitoring programmes. Fish biomass and biogenic habitat information were collected using standardized underwater visual census (UVC) methods based around a 50 m transect line placed by divers along a depth contour. Reef survey transects were conducted at 1109 sites across temperate Australia (south of 29° S) in water depths less than 25 m (figure 2). A site encompassed all reef surveys conducted within a 200 m diameter area, other than when a major geographical feature subdivided the transects (e.g. transects located either side of a headland with differing degrees of wave exposure).

#### (ii) Reef fish biomass

Reef fish biomass data were collected following the methods described in [35], with surveys consisting of two separate 5 m (wide) by 5 m (high) bands on either side of a 50 m transect line placed by divers along a depth contour. On all surveys, all reef fishes (Classes *Actinopterygii* and *Elasmobranchii*) in each band were identified to species, their abundances counted and their total length estimated to the nearest size category. Total length (defined as the length of the fish in cm from the tip of the snout to the end of the longer caudal fin) was binned into 2.5 cm size classes up to 15 cm, 5 cm size classes to 30 cm, 10 cm classes to 50 cm, and fishes over 50 cm binned into 12.5 cm size classes [35]. All skates and rays were removed prior to calculations of biomass due to uncertainty in their biomass estimates (see electronic supplementary material, table S1 for list of families removed from analysis).

**Table 1.** Variable importance (sum of weights) of each predictor from biogenic habitat, physical environment and anthropogenic model suites. Asterisks indicate predictors with a variable importance greater than or equal to 0.9, which were subsequently included in the global model suite.

category	predictor	variable importance (sum of weights)
biogenic habitat	turfing algae	1.00*
	sand	0.99*
	sessile invertebrates	0.96*
	sessile invertebrates * level of MPA protection	0.69
	understorey algae	0.36
	sand * level of MPA protection	0.35
	canopy algae	0.31
	turfing algae * level of MPA protection	0.16
	understorey algae * level of MPA protection	0.13
	canopy algae * level of MPA protection	0.04
physical environment	depth	1.00*
	currents	0.89
	relief	0.78
	currents * level of MPA protection	0.47
	wave exposure	0.44
	slope	0.38
	relief * level of MPA protection	0.28
	wave exposure * level of MPA protection	0.23
	depth * level of MPA protection	0.20
	slope * level of MPA protection	0.14
anthropogenic	level of MPA protection	1.00*
	human gravity	0.93*
	human gravity * level of MPA protection	0.90*
	distance from shore	0.59
	distance from shore * level of MPA protection	0.34

For each transect, biomass (kg) was estimated for each fish species by, first, correcting for diver bias in size estimation of individual animals [36], then applying length–weight relationships from FishBase [37] to abundance (counts) and corrected individual size estimates. Total biomass (per 500 m^2^) for each survey was then calculated as the sum of the biomass of all species observed in both bands and, prior to inclusion in all analyses, was log10(X + 0.001) transformed to reduce the influence of extremely high values.

#### (iii) Biogenic habitat data

For each reef fish survey transect, the state of the biogenic and abiotic reef habitat was also recorded by divers. To increase the spatial coverage of data for this study and capture trends across a broader number of MPAs, we combined biogenic reef habitat data collected through two different monitoring programmes that used different methods for quantifying habitat:

— Photo-quadrats (PQs) obtained through Reef Life Survey [35]. A series of photos taken by divers every 2.5 m along the 50 m transect and later scored to a set of morphological and functional groups in line with the CATAMI classification scheme [38] using a grid overlay of five points per photo, to a total of 100 points per transect.
— *In situ* quadrats obtained through the ATRC. Benthic habitat scored to species-level (or genus/family when required) or to general substrate categories (cobble, sand, etc.) by divers underwater using a 50-point quadrat placed at 10 m intervals along the transect. Three algal structural layers (canopy, subcanopy and substrate) were each assessed using the 50-point quadrats when present, allowing for a total maximum count per quadrat of more than 50 points or more than 100% habitat cover.

Due to differences in the two ways habitat data were recorded, the high-resolution, multidimensional *in situ* quadrat data were adjusted prior to analysis to mimic the two-dimensional data captured using PQs. To do so, each habitat category present within quadrats exceeding 50 points (i.e. greater than 100% cover) was scaled from the top down based on expert knowledge (authors: G.J.E., E.O. and R.S.-S.) of the structural composition of Australian temperate reef systems, until a total of 50 points or 100% cover was achieved, following the methods of [39].

Examination of the altered *in situ* data and PQ data showed similar temporal and spatial trends in all habitat groups considered, suggesting the data conversion was relatively robust in mimicking the information captured with a two-dimensional PQ, although there was limited spatial and temporal overlap in the two datasets (more information on validation provided in the electronic supplementary materials). Incomplete surveys were removed prior to analysis to ensure habitat coverage was representative of the whole transect.

The combined data (PQ and transformed *in situ* quadrats) were then aggregated to 16 coarse habitat categories based on those used in [25], and the percent cover of each category was averaged across each transect. These 16 coarse categories were then further aggregated into five categories that were more broadly comparable across our region of interest: percent cover of turfing algae, sessile invertebrates, sand, canopy-forming macroalgae (*laminarian* and *fucalean* kelps) and understorey-forming algae (foliose brown, green, red and calcified algae).

#### (iv) Physical environment data

Four physical environment covariates considered important for the provision of food, habitat and refuge on temperate reefs [17,40] were categorized post-survey at four levels for each reef site by divers familiar with the site:

— Wave exposure. The degree of oceanic swell a site receives on average: 1, sheltered; 2, maximum wave height 1−3 m; 3, ocean swell maximum greater than 3 m; 4, open swell from prevailing direction.
— Slope. Mean gradient along the transect line: 1, less than 1 : 10 gradient; 2, 1 : 10−1 : 4; 3, 1 : 4−1 : 2; 4, greater than 1 : 2.
— Relief. Mean vertical relief along the transect line: 1, less than 0.5 m; 2, 0.5–1 m; 3, 1–2 m; 4, greater than 2 m.
— Currents. Mean relative strength of the currents at a given site: 1, none; 2, weak; 3, moderate; 4, strong.

Mean depth of the transect line was also recorded by divers on all surveys and included as a physical environment covariate. The mean depth of all surveys was 6.5 m with a range from 1 to 25 m.

#### (v) Anthropogenic data

Three anthropogenic factors were estimated for each reef site and included in the predictor set to evaluate the role of human activity in shaping differences in fish biomass.

— Level of MPA protection. The level of MPA protection from fishing at time of survey. Surveys were categorized as either ‘fished’ where no MPA protection existed at time of survey; ‘partially protected’ where surveys were undertaken inside established MPAs with restricted fishing; and ‘fully protected’ where surveys were conducted in established MPAs where no fishing activities were permitted at time of survey.
— Human gravity. A measure of how large and far away a human population is to a given reef site, providing a relative indicator of the pressures associated with human population and activity. Human gravity was calculated as a function of population density within a 500 km radius of each reef site and approximate travel time (or accessibility) to the site, as detailed in [4]. Human gravity ranged from 0.57 to 34 856 across temperate Australia, with a mean value of 983.
— Distance from shore (m). The straight-line distance from each site to the nearest shoreline (in metres) was calculated using high-resolution shapefiles of the Australian coastline and a distance function in R to capture the influence of relative isolation of each reef site from human activity (i.e. a measure of accessibility). Mean distance from shoreline to site across all sites was 2,814 m and varied from 1 m to 60 447 m.

### (b) Statistical analysis

#### (i) Accounting for broad-scale gradients in reef fish biomass

As reef fish biomass has been shown to vary across broad spatial scales [6], with the potential to confound the local effects of biogenic habitat, physical environment and anthropogenic factors, we first built a random forest (RF) model [39] using a dataset of broad-scale environmental conditions and reef fish biomass measured on openly fished reefs (i.e. excluding data from sites surveyed inside MPAs) to predict expected biomass for the individual shallow temperate Australian reef sites investigated in this study. Biomass predictions provided a benchmark for comparing observed biomass values across our temperate Australian study area, with the ratio of observed biomass to predicted biomass used as the response variable in our main analysis. Our modelled predictions of site-level biomass in the absence of MPA protection enabled us to explore relative differences in MPA effects across broad spatial scales and in relation to local conditions. Alternative causal inference approaches, such as before-after-control-impact (BACI) designs (e.g. [41]), may provide more confidence in effect size estimates for each MPA, but unfortunately such monitoring designs were not available across all the MPAs studied here. In the absence of consistent ‘before’ data for most MPAs, we undertook an informal comparison of the counterfactual approach we used against a control/impact design for estimating MPA effect sizes, using all sites within a 20 km radius outside MPAs. This showed that effect sizes were largely similar (*r* = 0.68) between the two methods (see electronic supplementary materials). RF models were chosen to predict biomass across temperate Australia as they cope well with relatively small sample sizes, complex nonlinear relationships and strong collinearity between high numbers of categorical and continuous predictor variables [42,43].

The predictive RF model was built using the latest available reef fish biomass data from 724 openly fished reef sites across temperate Australia (figure 2). Of these sites, the RF model was trained on a random selection of 75% (*n* = 543) of all sites and tested on the other 25%. Only the latest survey data available for each site were used, with data spanning the period 2008–2022 inclusive. Although a single biomass snapshot at a given site may introduce noise due to short-term changes in environmental conditions (e.g. heatwaves), our model aims to capture broad-scale trends in biomass driven by long-term averages rather than conditions at the time of survey. We used only openly fished sites (‘fished’ protection level) outside of MPA boundaries in the model to avoid confounding biomass predictions with the effects of fishing protection.

Site latitude, longitude and 11 ocean environmental variables representing broad-scale ocean conditions, such as SST and primary productivity, extracted for each reef site coordinate were included as predictors of (log) biomass in a regression RF model built with replacement (bootstrapped). SST data were obtained from the National Oceanographic and Atmospheric Administration (NOAA) Coral Reef Watch programme’s global daily SST product modelled at a 5 km (0.05°) resolution [44]. Daily SST was first calculated for the two calendar years leading up to the latest available survey date at each reef site, before averaging across the 2 years to produce a site-level mean. The remaining environmental variables were extracted from Bio-Oracle [45,46] and represented the mean value at a reef site between 2000 and 2020 at the ocean surface (see electronic supplementary material, table S2 for full list of variables included). Bio-Oracle environmental variables were extracted for each of the site coordinates from global rasters with a native spatial resolution of 5 arcminutes (approximately 9.2 km at the equator; [41,42]). As RF models are relatively insensitive to multiple collinearities between predictor variables [42], several highly correlated predictors were retained in the model (e.g. SST mean and latitude). The RF model was built using the ‘ranger’ engine in the *Tidymodels* package in R [47].

Three key hyperparameters of the RF model were tuned over a series of folds in R to optimize model performance and estimates of variable importance: ntrees (number of trees in the forest); mtry (number of randomly selected predictors at each node); and tree size (smallest node size for splitting or maximum number of terminal nodes [48]). Tuned hyperparameter values were selected to minimize root mean square error (RMSE) scores, and model performance was then assessed using out of bag (OOB) error rate and *R*^2^ values [42].

The final, tuned RF model was then used to predict (log) biomass at all reef sites surveyed across temperate Australia for which co-located data on biogenic habitat, physical and anthropogenic factors were available. Prior to calculations of response ratios, all observed biomass and habitat data recorded on 500 m^2^ transects at these reef sites were averaged across all transects surveyed on each site and date combination to reduce variability in the data associated with transect placement and depth on any given day. As with the training data, only the latest available survey data for each site were used. Log response ratios (LnRR) were calculated for each site and date combination as the mean observed log biomass minus the predicted log biomass. Our multi-model inference analysis (detailed below) therefore involved data for 922 shallow (less than or equal to 25 m) temperate Australian reef sites, including 194 sites in 60 fully protected MPAs/MPA zones and 219 sites in 66 partially protected MPAs/MPA zones (electronic supplementary material, figure S5). Biomass log response ratios were therein included as the response variable in all linear mixed-effects model (LMM) analyses.

#### (ii) The importance of local factors in marine protected area effectiveness

#### Multi-model inference and model selection

To determine the relative importance of local factors in influencing differences in observed biomass from those expected based on broad-scale conditions, log response ratios of observed to predicted biomass across temperate Australia were assessed as a function of local biogenic habitat, physical environment and anthropogenic factors in a suite of LMMs using a multi-model inference framework [49].

All predictor variables were standardized, and human gravity and distance from shore were log_10_ transformed prior to inclusion. Multiple collinearities among predictor variables were explored using Pearson correlation matrices, with none of the covariates included in the analysis found to be highly correlated (*r* < 0.5; electronic supplementary material, figure S6).

For each model suite (described below), all possible additive combinations of the predictors were fitted to the data. To account for potential spatial autocorrelation arising from sites clustered close together, a random intercept for 100 × 100 km grid cells was included in all models. This allows the model to capture unmeasured spatial variation among proximate sites that may share environmental or ecological characteristics. Spatial autocorrelation in model residuals was assessed using Moran’s *I* test. No significant autocorrelation was detected in the residuals of the top model, indicating that the inclusion of grid cell as a random effect effectively accounted for spatial structure in the data. Models were then ranked based on Akaike’s information criterion adjusted for small sample sizes (AICc; [45]), with top models selected as the most parsimonious model in the top model suite (ΔAICc ≤ 4) with the lowest AICc score [50,51]. Model goodness-of-fit was determined for each top model using marginal (variance explained by fixed effects only) and conditional *R*^2^ (variance explained by both fixed and random effects) values, which provide robust measures of the variance explained for mixed-effects models where traditional *R*^2^ values do not [52]. LMMs were fitted using the *lme4* [53] and *MuMIn* [54] packages in R version 4.3.0 [55].

#### Reducing predictors and fitting the global linear mixed-effects model

To avoid over-fitting the global model, three model suites were first tested separately for each grouping of local factors. These model suites were: (i) biogenic habitat: percent cover of canopy algae, understorey algae, turfing algae, sessile invertebrates and sand; (ii) physical environment: wave exposure, slope, currents, relief and depth; and (iii) anthropogenic: level of MPA protection, human gravity and distance from shore. To test for interactions between local factors and the level of MPA protection, interaction terms between all covariates and level of protection were included in each model suite. All possible additive combinations from each suite of variables were fit, and the relative importance of each variable was then determined using the sum of the Akaike’s weights for all models in which they appeared [56]. All predictors with a variable importance greater than or equal to 0.9 were included in the global model set, with this set of predictors herein referred to as the *reduced predictor set*.

All possible additive combinations of the reduced predictor set were then fit, and the top global model selected according to the methods detailed above. Partial effects for the global model were plotted from standardized model estimates to compare the strength and direction of relationships, and measures of model fit were calculated as detailed above.

## 3. Results

### (a) Accounting for broad-scale gradients in reef fish biomass

The RF model built from broad-scale environmental covariates explained 42% of variation (*R*^2^) in fish log biomass distributions on fished reefs across temperate Australia, with an OOB prediction error of 0.28. Model RMSE scores were minimized when the RF was built over 1275 regression trees, 13 randomly selected predictors at each node, and a minimum tree size of 30 nodes. The RF variable importance plot (VIP) showed mean SST to be the strongest predictor of reef fish biomass across temperate Australia (electronic supplementary material, figure S7).

**Figure 3.**
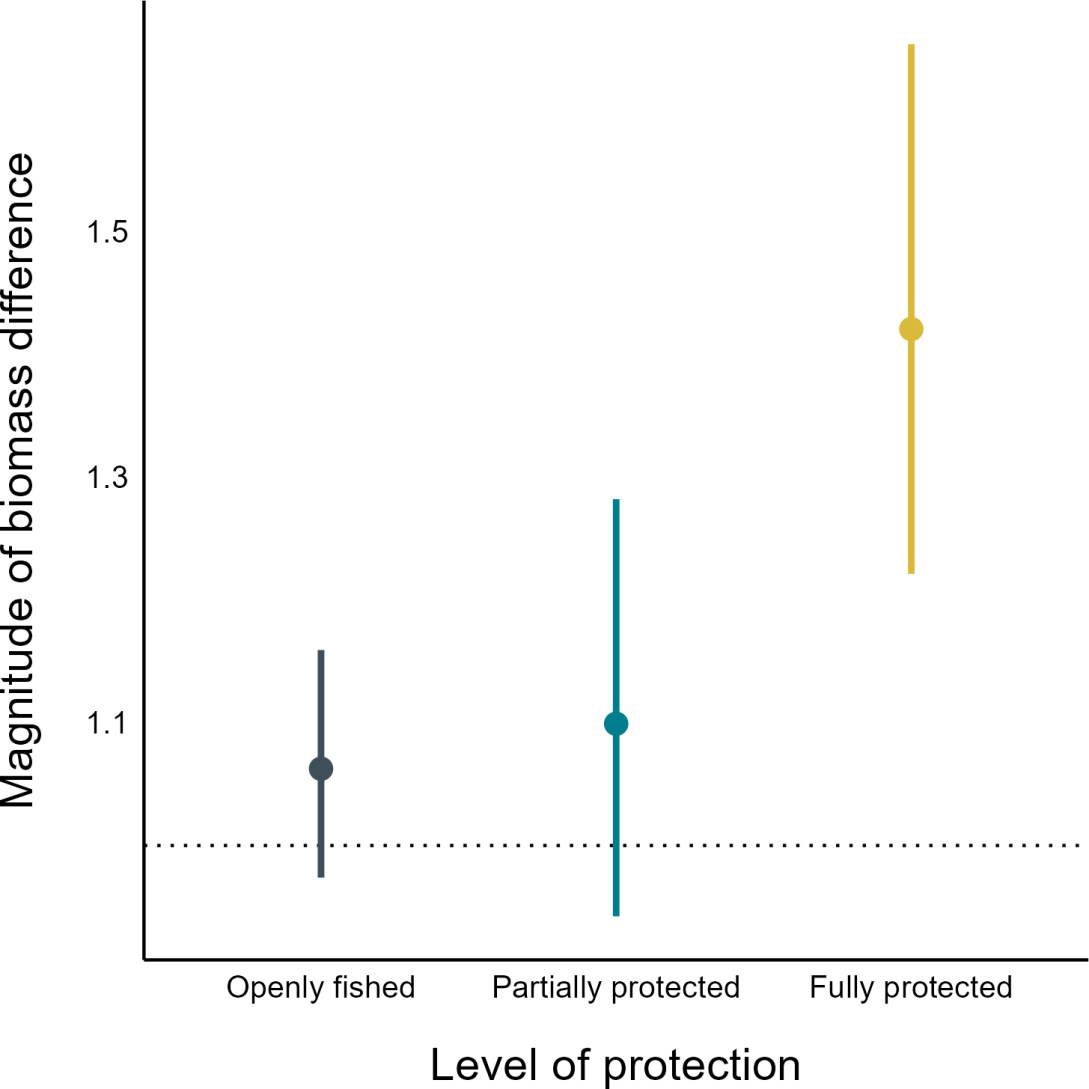
The mean difference between observed and predicted fish biomass across unprotected (dark blue), partially protected (light blue) and fully protected (yellow) temperate Australian reef sites, with 95% confidence intervals (CIs). The thin dotted horizontal line at 1 indicates the average prediction for fished reef sites in temperate Australia according to the RF model estimates.

After calculating response ratios based on RF predictions across each temperate reef site, the mean log_10_ response ratio was 0.06 ± 0.02 s.e.m. (standard error of the mean), which indicates that fish biomass recorded on surveys across temperate Australia is slightly higher (114% ± 5% s.e.m.) than the biomass expected based on environmental conditions. When averaged across each level of protection, however, response ratios revealed a clear split in mean response ratios. Unsurprisingly, fish biomass at openly fished sites was, on average, slightly higher but not significantly different to that predicted on fished coastlines across the region based on broad-scale environmental conditions (i.e. observed biomass was approximately 106% of predicted biomass, on average; figure 3). Similarly, inside partially protected areas, response ratios were not significantly different to openly fished sites (determined by visual inspection of the overlaps in 95% confidence intervals between levels of protection; figure 3) and, in fact, were similar to the average on openly fished coastlines (i.e. mean response ratio was not significantly different to 1). On the other hand, response ratios inside fully protected MPAs were significantly higher than those at openly fished sites, with observed fish biomass, on average, 34% greater than the mean biomass at unprotected sites across temperate Australia. On average, response ratios inside fully protected MPAs were higher than those in partially protected MPAs, but not significantly so.

### (b) The importance of local factors in marine protected area effectiveness

#### (i) Reducing predictor variables from models

Based on variable importance from the separate biogenic habitat, physical environment and anthropogenic model suites, understorey algae, canopy algae, currents, relief, wave exposure, slope, human gravity and distance from shore were excluded from the final predictor set (sum of AICc weights in table 1). Interaction terms between local covariates and level of MPA protection were also excluded due to low support (sum of weights less than or equal to 0.9), except for the interaction between MPA protection and human gravity. Thus, habitat effects were consistent in openly fished, partially protected and fully protected locations. The final reduced predictor set for assessment in the global LMMs therefore included the fixed effects of turfing algae, sand, sessile invertebrates, depth, level of MPA protection, human gravity and the interaction between human gravity and MPA protection.

### (c) Fitting the global linear mixed-effects model

Out of all possible additive combinations of the reduced predictor set, the most parsimonious model with the lowest AICc in the top model set included the fixed effects of level of MPA protection, depth and the percentage cover of turfing algae and sand. The goodness-of-fit for this top model (marginal *R*^2^ = 0.07, conditional *R*^2^ = 0.16) was slightly improved relative to the top model from the separate biogenic habitat (marginal *R*^2^ = 0.04, conditional *R*^2^ = 0.12), physical environment (marginal *R*^2^ = 0.05, conditional *R*^2^ = 0.15) and anthropogenic (marginal *R*^2^ = 0.03, conditional *R*^2^ = 0.12) model suites.

**Figure 4.**
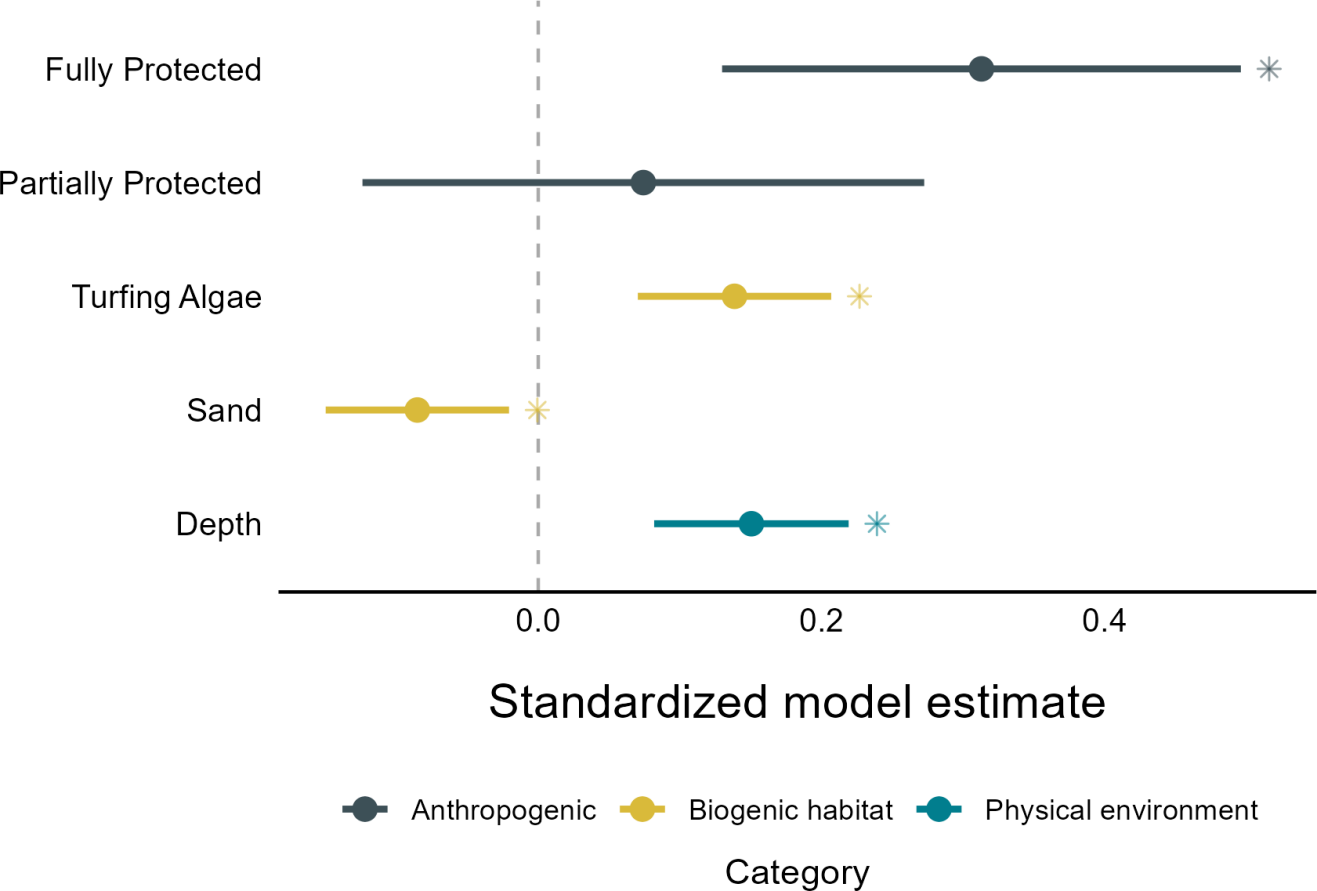
Standardized model estimates from the top model based on all possible combinations of the reduced predictor set. Asterisks indicate significant relationships with the response variable (fish biomass log response ratio).

When applied to the full dataset, all predictor variables included in the top mode were found to have significant effects, with the exception of partial protection which exerted a positive but non-significant effect on response ratios (figure 4). After accounting for the random effect of spatial grid cell, full MPA protection had the strongest impact on response ratios across temperate Australia, exerting a significant positive influence. Although the influence of full MPA protection was stronger than that of partial protection, with a mean standardized model estimate more than four times higher, their relationship with response ratios was not significantly different.

Based on the global model, strong positive relationships with local response ratios indicate greater than expected fish biomass at temperate reef sites with greater depth or percent cover of turfing algae. Alternatively, reef sites with greater cover of sand were found to have lower fish biomass than expected, as indicated by significant negative influences on local response ratios.

## 4. Discussion

We used a global model of total fish biomass along openly fished coastlines and 11 broad-scale environmental variables to predict fish biomass at 922 temperate reef sites across a large network of 49 MPAs in temperate Australia. Our results suggest that fish biomass inside fully protected MPAs across this network is 34% greater, on average, than predicted for openly fished sites in the region. In contrast, fish biomass inside partially protected MPAs across the network was not significantly different to predicted for openly fished coastlines (110% of predicted biomass, on average). These results demonstrate the importance of fishing pressure in determining distributions of reef fish biomass across temperate Australia and are consistent with a substantial body of research showing ecological outcomes are generally greater in MPAs with strict prohibitions on fishing (i.e. no-take [31,57]).

However, while biomass in partially protected areas may approximately average to levels seen in fished coastlines elsewhere, the difference between observed and predicted biomass across the 66 partially protected MPA zones from 21 MPAs in this study may also be influenced by factors such as their design, management and enforcement. Level of protection against fishing is considered one of the most important factors for driving fish biomass gains inside MPAs [7,58,59]. While no-take protection appears to consistently generate better biodiversity outcomes than partial protection, fish biomass gains inside partially protected MPAs have often been demonstrated [60]; nevertheless, results are inconsistent and most likely to occur with a subset of the commercially and recreationally important fish species [61]. Across the partially protected MPAs included here, restrictions on fishing varied from minimally protected areas that permit all commercial and recreational fishing, to areas that permit only recreational fishing, and more restrictive areas that only allow certain gear types or species. The partially protected MPAs also span a range of ages (from under 5 years old to over 30 years) and sizes (from less than 1 km^2^ to over 1000 km^2^) which may further influence the differences in biomass in these areas compared with openly fished coastlines [6]. The heterogeneity in response ratios observed in partially protected areas probably reflects the balance between biomass gains in larger, older and/or more heavily restrictive partially protected areas, and negative or neutral effect sizes in smaller, younger and/or less restrictive areas.

To determine the relative importance of local factors and protection on biomass across our study region, we then used a multi-model inference approach to relate biomass response ratios to biogenic habitat, physical environment and anthropogenic conditions at the reef site. Again, fishing pressure was shown to have the strongest influence on differences in fish biomass, with full protection exerting the largest influence out of all covariates considered, but local biogenic habitat and physical environment also significantly influenced the size of response ratios across all levels of protection. Cover of turfing algae and depth exerted significant positive influences on response ratios inside the MPA network, with higher values of each associated with greater biomass than predicted for the environmental and management context of the reef. Alternatively, the percent cover of sand had a significant negative influence on biomass across all sites. Thus, while strict protection from fishing appears to be the most important factor for securing positive ecological outcomes in temperate Australian MPAs, local biogenic habitat and physical environmental characteristics play an important role in shaping the magnitude of biomass recovery relative to nearby fished coastlines.

Notably, no clear interaction was evident for any habitat effect in either fished/partially protected or fished/fully protected zone comparisons. Thus, the effects of habitat on fish biomass were consistent regardless of level of protection. This finding simplifies zone comparisons in an era of rapid habitat change [62], given that consistent regional changes in habitat type that extend across management zones should not influence zone relativities in fish biomass observations. However, increases in, for example, turfing algae in fished zones relative to protected zones will affect estimates of differences associated with MPAs.

Interestingly, across all levels of protection, higher coverage of turfing algae was associated with greater-than-expected fish biomass. This is surprising, given that turfing algae are often associated with degraded reef states and lower food, habitat and productivity compared with macroalgae [63,64], although turf appears to support greater epifaunal secondary productivity in temperate reef systems [65]. One possible explanation for this unexpected result could be biases in the UVC methods used to collect biomass data, where canopy cover and diver shyness might affect fish detectability and impact biomass estimates [36]. However, kelp removal experiments have shown little impact of algal cover on visual biomass estimates in temperate Australia [66], and the consistent positive relationship with turf across all levels of protection suggests differences in diver shyness in response to fishing pressure is not the underlying cause of this outcome [67].

Alternatively, this counter-intuitive relationship may reflect how habitat characteristics shape reef communities from the bottom up, with implications for the magnitude of MPA effects. For example, in kelp-dominated areas, dense canopy cover may provide ambush predators with partial refugia, leading to smaller fish aggregations. In contrast, areas of low canopy cover (e.g. turf-dominated reefs) may expose predators, reducing predation risk and allowing greater fish aggregation and biomass. This may be particularly true for planktivorous fishes, which are not directly reliant on biogenic habitat for food, and constitute the majority of biomass on shallow temperate Australian reefs [68]. Accordingly, we might expect higher planktivore biomass on low-profile habitats, where predation pressure is lower, leading to greater total biomass and MPA effects overall. To explore this further, we compared total biomass across fully protected sites and found turf-dominated sites (turf greater than canopy) consistently supported higher biomass than canopy-dominated sites in most MPAs (electronic supplementary material, figure S8). These findings highlight the importance and complexity of local habitat structure in shaping reef communities and reinforce the need to consider ecological context in MPA evaluations.

Here we demonstrate that local reef conditions can significantly influence the recovery of fish biomass within a large MPA network. However, the design, placement and management of large MPA networks often overlook the differences in underwater conditions across these areas, their impact on marine communities and the resulting success or failure of the MPAs both in the short and long term. Nevertheless, our findings suggest that conservation outcomes could be improved by adjusting MPA design, placement or management to optimize conditions that promote community recovery. In degraded temperate reef areas, for example, additional interventions that restore the biogenic habitat first, such as removing or controlling problematic species [69], could be crucial for promoting bottom-up community recovery, ultimately leading to better long-term conservation outcomes. Implementing targeted and well-researched strategies could drastically transform the effectiveness of individual MPAs and large MPA networks, ensuring they are not only protecting reef biodiversity but also actively fostering the recovery of natural communities. Consideration of conditions across the broader seascape that influence fish biomass recovery could also help to refine expectations of conservation outcomes inside MPAs.

## 5. Conclusions

Our study highlights the critical role that fishing plays in shaping the state of shallow coastal reef ecosystems across broad spatial scales, and the importance of strict fishing prohibitions in MPAs for enhancing fish biomass and achieving positive ecological outcomes. We found that fully protected MPAs consistently supported higher fish biomass than if fishing was allowed, underscoring the importance of full protection measures for achieving maximum biodiversity outcomes. However, the influence of local habitat characteristics, such as depth and biogenic habitat types, also emerged as significant factors that shape the magnitude of biomass recovery across the MPA network. This indicates that while protection from fishing is paramount, the specific environmental context of each reef can also influence conservation success.

Overall, our results emphasize the need for MPA networks to be designed and managed with a nuanced understanding of local reef conditions. By incorporating habitat characteristics into MPA planning, management and monitoring, and by considering the broader seascape context, expectations of biomass recovery can be defined. This will allow for more effective and targeted conservation efforts, and critically, effective monitoring of MPA policy impact (e.g. are the MPAs doing what we are expecting them to do). As climate change and human activities continue to alter reef habitats, strategically positioned, well-managed and effectively monitored MPAs are critical in the preservation of marine ecosystems and ensuring their resilience in the face of ongoing environmental changes.

## Data Availability

The datasets generated and analysed during this study are available from Dryad at [70]. Supplementary material is available online [71].
